# Potential Gut Microbiota Features for Non-Invasive Detection of Schistosomiasis

**DOI:** 10.3389/fimmu.2022.941530

**Published:** 2022-07-14

**Authors:** Datao Lin, Qiuyue Song, Jiahua Liu, Fang Chen, Yishu Zhang, Zhongdao Wu, Xi Sun, Xiaoying Wu

**Affiliations:** ^1^ Department of Parasitology, Zhongshan School of Medicine, Sun Yat-sen University, Guangzhou, China; ^2^ Key Laboratory of Tropical Disease Control, Ministry of Education, Guangzhou, China; ^3^ Chinese Atomic Energy Agency Center of Excellence on Nuclear Technology Applications for Insect Control, Provincial Engineering Technology Research Center for Diseases-Vectors Control, Guangzhou, China; ^4^ Department of Clinical Laboratory, Xiangyang No.1 People’s Hospital, Hubei University of Medicine, Xiangyang, China; ^5^ School of Medicine, South China University of Technology, Guangzhou, China; ^6^ The Third Affiliated Hospital, Sun Yat-sen University, Guangzhou, China

**Keywords:** *Schistosoma japonicum*, schistosomiasis, parasites, gut microbiota, non-invasive

## Abstract

The gut microbiota has been identified as a predictive biomarker for various diseases. However, few studies focused on the diagnostic accuracy of gut microbiota derived-signature for predicting hepatic injuries in schistosomiasis. Here, we characterized the gut microbiomes from 94 human and mouse stool samples using 16S rRNA gene sequencing. The diversity and composition of gut microbiomes in *Schistosoma japonicum* infection-induced disease changed significantly. Gut microbes, such as *Bacteroides*, *Blautia*, *Enterococcus, Alloprevotella*, *Parabacteroides* and *Mucispirillum*, showed a significant correlation with the level of hepatic granuloma, fibrosis, hydroxyproline, ALT or AST in *S. japonicum* infection-induced disease. We identified a range of gut bacterial features to distinguish schistosomiasis from hepatic injuries using the random forest classifier model, LEfSe and STAMP analysis. Significant features *Bacteroides*, *Blautia*, and *Enterococcus* and their combinations have a robust predictive accuracy (AUC: from 0.8182 to 0.9639) for detecting liver injuries induced by *S. japonicum* infection in humans and mice. Our study revealed associations between gut microbiota features and physiopathology and serological shifts of schistosomiasis and provided preliminary evidence for novel gut microbiota-derived features for the non-invasive detection of schistosomiasis.

## Introduction

Schistosomiasis, one of the most important human parasitic diseases, affects over 240 million people and threatens nearly one-eighth of the world population at risk of infection worldwide (1–3). This zoonotic disease is caused by trematodes of the genus *Schistosoma* including *Schistosoma japonicum* (*S. japonicum*), *Schistosoma mansoni*, *Schistosoma haematobium*, *Schistosoma intercalatum* and *Schistosoma Mekongi* (4, 5). In China, *S. japonicum* is the only endemic parasitic flatworm of schistosome. Among the 12 province endemics for schistosomiasis in China, Yunnan, Hubei, Anhui, Jiangxi and Hunan provinces maintained the criteria of transmission control by the end of 2020 (6). There are still 29,517 advanced schistosomiasis cases documented (6), implying that *S. japonicum* infection remains one of the most important public health problems in some endemic areas of China. To achieve the goal of the *National Thirteenth Five Year Plan*, strategies for schistosomiasis control are still required.

The lifecycle of *S. japonicum* includes the several developmental stages of paired adult worms, eggs, ciliated miracidium, mother and daughter sporocyst and cercariae (1, 4). Immature schistosomula migrate downstream to the hepatic portal-mesenteric system. Then, females become mature in the liver and release thousands of eggs. The eggs retained in host tissues can induce hepatic granuloma formation, which ultimately evolves into progressive hepatic fibrosis and portal hypertension in advanced schistosomiasis patients (1, 5, 7). Despite the wide use of praziquantel (PZQ) for the prevention and control of schistosomiasis in endemic regions, hepatic fibrosis remains a serious chronic schistosome-infected disease affecting public health (5, 8). Until now, liver biopsy is the gold standard for the assessment of fibrosis, but the disadvantages such as poor patient compliance, morbidity, mortality and sampling error limited the wide application of liver biopsy (9–11). Therefore, a comprehensive evaluation of the stage of liver fibrosis is still essential. Previous studies have revealed that serum tests (such as aspartate aminotransferase (AST), alanine aminotransferase (ALT) and miR-146a-5p) (9, 12, 13) and liver stiffness measurement (LSM) (11, 14) are some important diagnostic indexes of liver fibrosis. Ultrasonography has been used for the assessment of hepatic fibrosis of advanced schistosomiasis but it showed only a moderate correlation between Ultrasonography and liver biopsy (15, 16). However, the limitations and costs of these invasive indexes and imaging detection restrict their widely used for the detection of liver fibrosis. Thus, research on more non-invasive tools for the prediction of the level of *S. japonicum* infection-induced liver fibrosis is still essential.

Mammals harbor hundreds of gut microbiota that affect host biology and health (17). Recent studies have demonstrated that *Schistosoma* infection can affect the composition and structure of gut microbiota in infected patients (18, 19) and mice (20, 21). Previous research suggested that gut microbiota composition is associated with *S. mansoni* infection burden in rodent models (22). These studies indicated that *Schistosoma* infection is associated with the diversity and composition of gut microbiota in infected mammals. The liver-gut microbiota axis is involved in the physiopathology of liver diseases, implying that gut microbiota may be associated with fibrotic development or progression (23, 24). Evidence suggests that gut microbiota, such as *Veillonellaceae* and *Ruminococcaceae*, are reliably predicted biomarkers for the detection of hepatic fibrosis and damage (25–27). However, the relationship between the gut microbiota and hepatic fibrosis induced by *S. japonicum* remains unclear. Accordingly, whether gut microbiota alterations in *S. japonicum* infection-induced disease can be used as potential non-invasive biomarkers for the detection of the level of hepatic fibrosis is unclear.

In this study, we hypothesized that a strong relationship exists for the development of gut microbiota features derived biomarkers that can be used to predict the hepatic fibrosis and granuloma in *S. japonicum* infection-induced diseases. Here, we comprehensively investigated the relationship among gut microbiomes, serologic detection and the level of hepatic injuries in *S. japonicum*-infected mice, and compared it with the gut microbiota of human individuals. We aimed to develop a panel of gut-microbiota-derived biomarkers for the non-invasive detection of the level of hepatic injuries in schistosomiasis.

## Materials and Methods

### Mice

We purchased 56 male pathogen-free BALB/c mice (6-week-old, 18 ± 2 g) from the Experimental Animal Center of Southern Medical University. The mice were reared in plastic cages with free access to autoclaved sterile water and chow in the Biosafety Level-2 (BSL-2) laboratory of Sun Yat-Sen University. The rearing conditions include controlled temperature and humidity and a 12 h light and 12 h dark cycle. We randomly divided the mice into groups for further experiments.

### *S. japonicum* Cercariae and Infection

The *S. japonicum* cercariae were obtained from infected *Oncomelania hupehensis*, which were purchased from the Chinese Center for Disease Control and Prevention (Shanghai, China). The steps of releasing cercariae from snails were conducted described as in a previous study (28). The mice were infected with 5, 15, 20 or 60 *S. japonicum* cercariae per individual *via* the shaved abdominal skin, and treatment was continued for 7 or 8 weeks. The mice uninfected were served as the control group.

### Sample Collection and Detection From Mice

All mice were sacrificed after chloral hydrate asphyxiation and cervical dislocation at 49 or 56 days after infection (dpi) of *S. japonicum*. Luminal content samples were collected from colon intestines at 49 or 56 dpi under sterile conditions and transferred directly into sterile tubes. Left liver lobes and colons were collected and immediately fixed in 4% paraformaldehyde for the histopathological analysis described in the previous study (29). Liver samples were also collected for the detection of hydroxyproline content according to the protocol of the hydroxyproline assay kit (Nanjing, China). Blood samples were drawn from orbital veins and centrifuged at 1,500 g for 15 min. We then collected the serum samples after clotting for ALT and AST measurements in KingMed Diagnostics (Guangzhou, China). These samples were snap-frozen on dry ice and stored at -80°C until further study.

### Histological Staining

The fixed fragments from the liver were sliced into sections for histopathological analysis. These paraffin slices were dewaxed for the Masson’s trichrome staining and hematoxylin and eosin (H&E) staining. We captured the images of staining slices using an inverted microscope (Japan). We analyzed the percentage of the granulomatous and fibrotic area using a ZEISS Axio Scan automated slide scanner microscope (Germany). We also obtained a full view of the whole liver tissue. We analyzed the area of the whole tissue and the blue positive region using Image-Pro Plus 6.0 software (Media Cybernetics, USA). We calculated the percentage of the granulomatous and fibrotic areas described in the previous study (29).

### Subjects and Sample Collection From Humans

Twenty-six participants (with *S. japonicum* infection or without) of the previous study (30) were more than 30 years old, and they were initially screened for *Schistosoma* spp. eggs in feces using the Kato-Katz method (30, 31), and finally, twenty-six stool samples (15 controls, 11 patients) were colllected (30). To further investigate specific diagnostic biomarkers of schistosomiasis, we analyzed the association between shifts of gut microbiota of humans infected with non-parasitic factors (Such as hepatitis B virus) and the level of liver injuries. To this end, we recruited participants, and all participants from the Third Affiliated Hospital of Sun Yat-sen University were included in the study. All these participants from the Third Affiliated Hospital were >30 years old with non-parasitic factor infection, and the patients display clinical symptoms of liver injuries, and second liver two half-and-half detect provided reliable clinical test results of the participants (**Supplementary Table S1**). The information of participants about age, sex, diagnosis, medication, length of infection, HBsAg, HBsAb, HBeAg, HBeAb, HBcAb was shown in **Supplementary Table S1**. The participants were diagnosed with Hepatitis B virus-induced liver cirrhosis, and a total of twelve fresh fecal samples were collected.

### Liver Stiffness Measurement in Humans

Twelve patients with cirrhosis from the Third Affiliated Hospital were included in the present study. All participants were >30 years old with non-parasitic factor infection and the patients displayed clinical symptoms of liver injuries (Clinical information was shown in **Supplementary Table S1**). The participants were subjected to two kind of Liver stiffness measurements: A two-dimensional shear wave elastography (2D SWE) examination and transient elastography which performed using FibroTouch, and the measurements were performed according to the operations manual as described in the previous study (32–35). Twelve fresh fecal samples were collected and stored in Eppendorf tubes at –80°C. All participants gave informed written consent.

### DNA Extraction, PCR amplification and Sequencing

Total DNA from fecal samples was isolated using the protocol of the Hipure Stool DNA Kit (Guangzhou, China). After extraction, we detected the DNA quality and quantity examination using NanoDrop 2000 spectrophotometer (USA). The 16S rRNA gene (for V3-V4 regions) from mice gut microbiota was amplified using a bacterial primer set: 338F 5’-ACTCCTACGGGAGGCAGCA-3’ and 806R 5’- GGACTACHVGGGTWTCTAAT-3’. We performed the PCR amplification using the Takara PrimeStar DNA polymerase (Dalian, China). The following PCR cycling conditions included: denaturation at 95°C for 5 min, 25 cycles of 95°C for 30 s, 50°C for 30 s, 72°C for 40 s, and final extension at 72°C for 5 min. The PCR products were analyzed using 2% agarose gel electrophoresis. An amplicon library for sequencing was generated from the individual specimens. Finally, all fecal samples were sequenced on an Illumina HiSeq 2500 platform constructed by Biomarker technologies (Beijing, China).

### Microbiome Analysis

After the base calling analysis, we transformed the raw data files from the sequencing platform and NCBI (30) into the original sequenced reads and stored them in FASTQ format. QIIME (version 1.8.0) was used to cluster reads into operational taxonomic units (OTUs) and identified at 97% or more similarity (36). To analyze the alpha diversity, we rarified the OTU table using mothur (v. 1.33.3) (37). PERMANOVA and analysis of similarities (ANOSIM) were used to evaluate the beta diversity using mothur. The partial least squares discrimination analysis (PLS-DA), Statistical analysis of metagenomic profiles (STAMP) and principal coordinates analysis (PCoA) graphs (based on Bray Curtis distance) were done in R software (v. 2.15.3). Taxonomic characterization was performed using the linear discriminant analysis (LDA) effect size (LEfSe). Based on the genus or OTUs abundance of gut microbiota of mice, we conducted random forest algorithms with 500 random permutations described in the previous study (38). The relationships among variables were analyzed using Spearman analysis in R. The area under the receiver-operating characteristic (ROC) curves (AUC) was measured using GraphPad Prism (v. 6.0; USA). For range adjustment, all pairwise comparison between two groups was tested using *Wilcoxon*-test.

### Overview of the Sequencing Information Gut Bacterial Samples

Finally, a total of 94 bacterial samples obtained from sequencing and NCBI were investigated *via* 16S rRNA gene sequencing. We generated a total of 5,731,918 clean reads from these samples, with an average of 60,977 reads obtained from each sample. After being processed and filtered, over 800 and 504 operational taxonomic units (OTUs) with 97% minimum identity were annotated from human and mouse sequences, respectively.

### Statistical Analysis

Data are expressed as the mean ± standard error of the mean (SEM). The differences between groups were analyzed by student’s *t*-test using SPSS 19.0 software (USA). ^*^ *P* < 0.05, ^**^ *P* < 0.01 and ^***^ *P* < 0.001 were considered statistically significant.

## Results

### Distinct Signatures of Gut Microbiomes in *S. japonicum*-Infected Mice With the One-Time Point

**Figure 1:**
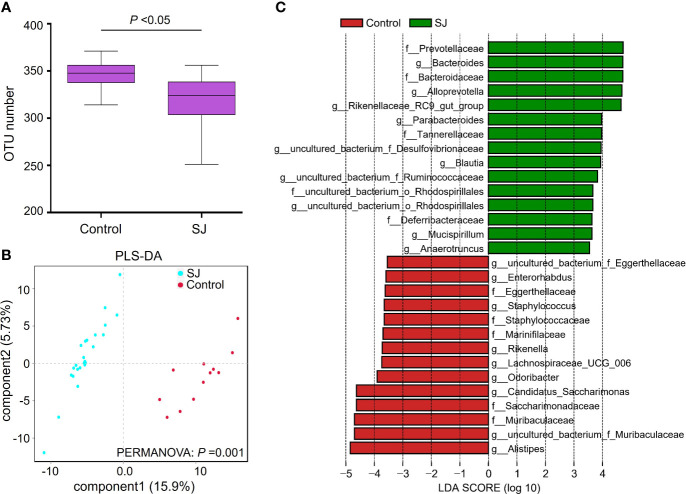
Comparisons of gut microbiota between *S. japonicum-*infected (n = 24) and uninfected (n = 12) mice. **(A)** OTUs analysis. **(B)** PLS-DA analysis. **(C)** Differential gut bacterial taxa were analyzed by LEfSe analysis with LDA score >3.5 between groups. Control: without *S. japonicum* infection mice. SJ: *S. japonicum-*infected mice. *P < 0.05 indicates significant difference.

The previous study has revealed that *S. japonicum* infection could induce alteration of gut microbiota in mice (29). In this study, *Bacteroidetes* and *Firmicutes* were the dominant phyla in mice (**Figure S1A**). The top 20 genera of gut microbiota in mice were shown (**Figure S1B**). We found significantly different gut bacterial diversities (based on OTU number) and community structures (based on PLS-DA) in *S. japonicum-*infected mice (**Figures 1A, B**, **S1**), regardless of the treatment on mice. In addition, a range of gut bacterial taxa, such as *Bacteroides*, *Blautia* and *Bacteroidaceae*, were considered as biomarkers to distinguish infected and uninfected mice using LEfSe analysis (**Figures 1C**, **S1**).

### Distinct Signatures of Gut Microbiomes at the One-Time Point With Different Numbers of *S. japonicum* Infection

Alteration of gut microbiota in mice with varied *S. japonicum* infection is unclear. We found that gut microbiota community structure (based on PCoA) in varied *S. japonicum-*infected mice differed significantly, which was calculated by PERMANOVA analysis based on Bray Curtis distance (**Figures S2A, B**). There was a significant difference in gut bacterial diversities (based on the Simpson index) between infected and uninfected mice (**Figure S2C**). The top 20 genera of gut microbes in mice showed a higher relative of *Bacteroides*, *Alistipes* and *Blautia* in infected mice (**Figure S2D**).

### Distinct Signatures of Gut Microbiomes at Different Time Points With Different Numbers of *S. japonicum* Infection

**Figure 2:**
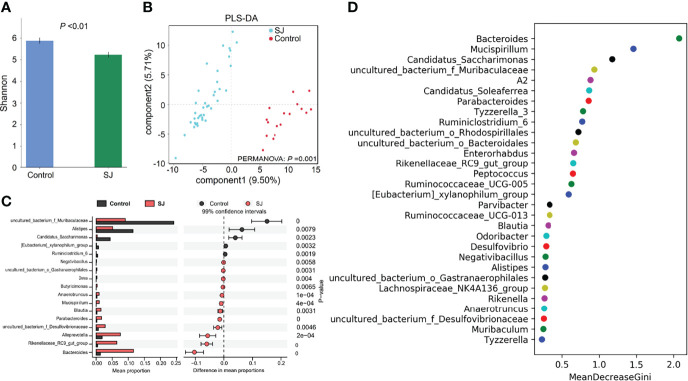
Taxonomic differences of gut microbiota between *S. japonicum-*infected (n=39) and uninfected (n = 17) mice. **(A)** Shannon index. **(B)** PCoA plot. **(C)** The difference of gut microbes in mice at the genus level using STAMP. **(D)** Top 30 of different gut microbes between populations are shown . Control, without *S. japonicum* infection mice. SJ, *S. japonicum-*infected mice.

There were significant differences in gut bacterial diversities (based on Shannon index) and community structures (based on PLS-DA; PERMANOVA, *P* =0.001) between *S. japonicum-*infected and uninfected mice (**Figures 2A, B**). At the genus level, a higher abundance of gut microbes such as *Bacteroides*, *Alloprevotella* and *Blautia* but a lower abundance of gut microbes such as uncultured bacterium f *Muribaculaceae*, *Alistipes* and *Candidatus Saccharimonas* in *S. japonicum*-infected mice were found using Statistical analysis of metagenomic profiles (STAMP) (**Figure 2C**). In addition, thirty bacteria, such as *Bacteroides*, *Alistipes*, *Blautia* and *Desulfovibrio*, were considered as potential predictors for distinguishing *S. japonicum*-infected and uninfected mice based on a machine-learning strategy (**Figure 2D**). Similar results were found using LEfSe analysis (**Figure S3**).

To further investigate specific diagnostic biomarkers of schistosomiasis, we compared the difference in the gut microbiota of mice infected with non-pathogenic factors and pathogenic *S. japonicum*. Previous studies have shown that probiotic *Bacillus subtilis* could affect the composition of gut microbiota and the level of liver disease in mice (29, 39, 40). Here, we showed that gut microbiota diversity and structure of gut microbiota of mice treated with *Bacillus subtilis* (non-pathogenic factor) significantly differed from these of mice infected with *S. japonicum* using Shannon index (**Figure S4A**) and PLS-DA analysis (**Figure S4B**), and the diversity of gut microbiota of mice treated with *Bacillus subtilis* is significantly higher than that of *S. japonicum-*infected group. Based on the LEfSe analysis, a range of gut bacterial taxa, such as *Bacteroides*, *Blautia* and *Bacteroidaceae*, could be considered as potential biomarkers to distinguish among control, *S. japonicum-*infected and *Bacillus subtilis-*infected mice (**Figure S4C**). In addition, gut microbes, such as *Bacteroides* and *Blautia*, were considered as potential predictors for distinguishing *S. japonicum*-infected and *Bacillus subtilis-*infected mice based on a machine-learning strategy (**Figure S4D**) and LEfSe analysis (**Figure S4E**).

### Relationships Among Granulomatous and Fibrotic Area and Gut Bacterial Variables in Mice

**Figure 3:**
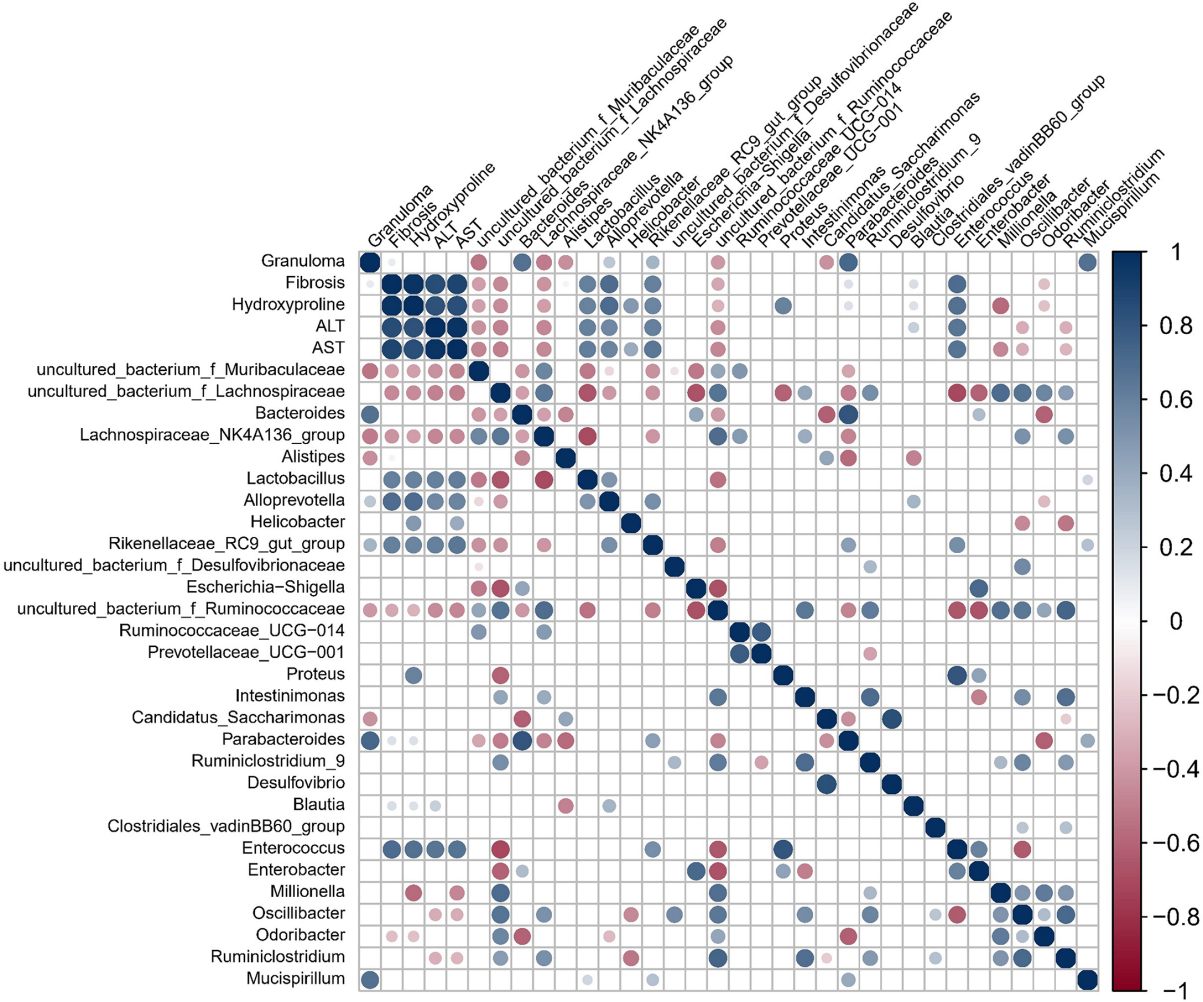
Spearman correlation for variables in *S. japonicum-*infected mice was shown. The circle size indicates the magnitude of the correlation. The circle in color indicates *P* <0.05, *P* < 0.01 or *P* < 0.001.

Granuloma showed a significant positive correlation with gut microbes *Bacteroides*, *Alloprevotella*, *Parabacteroides* and *Mucispirillum* but a significant negative correlation with Alistipes, *Lachnospiraceae* NK4A136 group, and Candidatus Saccharimonas (**Figure 3**). Fibrosis showed a significant positive correlation with Hydroxyproline, ALT, AST, *Blautia*, *Enterococcus*, *Lactobacillus*, *Alloprevotella*, *Parabacteroides* and *Rikenellaceae* RC9 gut group but a significant negative relation with uncultured bacterium f *Muribaculaceae*, uncultured bacterium f *Lachnospiraceae* and *Odoribacter*. Hydroxyproline, as well as ALT and AST, also showed a significant correlation with a range of gut microbes.

### Distinct Signatures of Gut Microbiomes Between *S. japonicum*-Infected and Uninfected Patients

**Figure 4:**
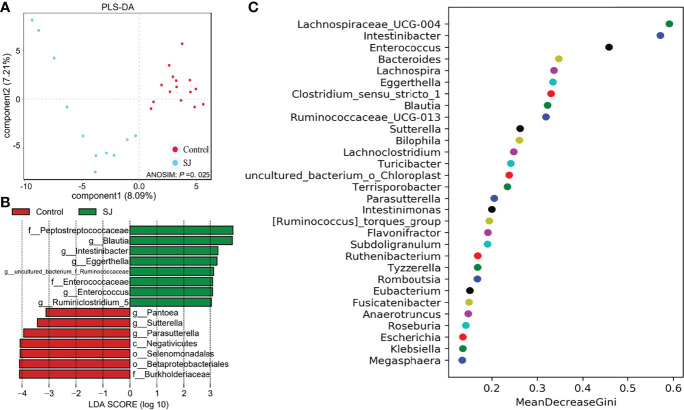
Identification of gut microbial biomarkers in *S. japonicum-*infected (n = 11) and uninfected (n = 15) humans. **(A)** PLS-DA plot. **(B)** Differential gut bacterial taxa analyzed by LEfSe analysis with LDA score >3 between groups. **(C)** Top 30 of different gut microbes between populations are shown. Control, without *S. japonicum* infection humans. SJ, *S. japonicum-*infected humans.

There were significant differences in gut bacterial community structures (based on PLS-DA; ANOSIM, *P* =0.025) between *S. japonicum-*infected and uninfected humans (**Figure 4A**). Gut microbes such as *Blautia* and *Pantoea* were considered as biomarkers to distinguish between infected and uninfected humans (**Figures 4B**, **S5**). Importantly, thirty gut bacterial genera, such as *Bacteroides*, *Blautia*, *Bilophila*, *Enterococcus*, *Intestinibacter* and *Desulfovibrio* were considered as a predictor for distinguishing *S. japonicum*-infected and uninfected humans based on a machine-learning strategy (**Figure 4C**). In addition, thirty gut microbes at the family level for distinguishing *S. japonicum*-infected and uninfected humans were shown (**Figure S5C**).

### Gut Microbiota Features Reflected the Granulomas or Fibrosis of *S. japonicum* Infection in Hosts

**Figure 5:**
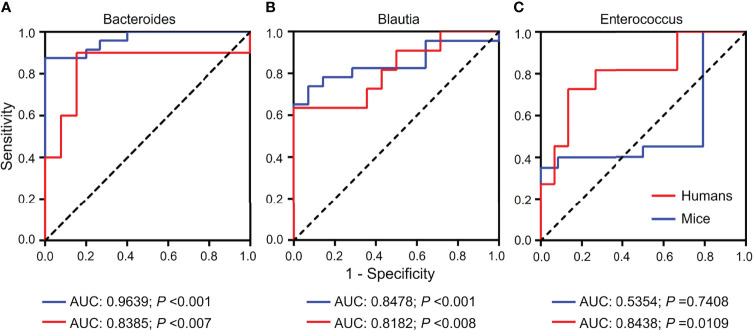
Receiver-operating characteristic (ROC) curves for the diagnosis of granulomas or fibrosis in *S. japonicum-*infected and uninfected hosts. ROC curves using three bacteria including *Bacteroides* **(A)**, *Blautia* **(B)** and *Enterococcus* **(C)** were plotted for the diagnosis. The area under the ROC curves (AUC) was calculated. The rate of identified outliers of *Bacteroides* is 10% and the rate of identified outliers of *Blautia* and *Enterococcus* is 5%.

**Figure 6:**
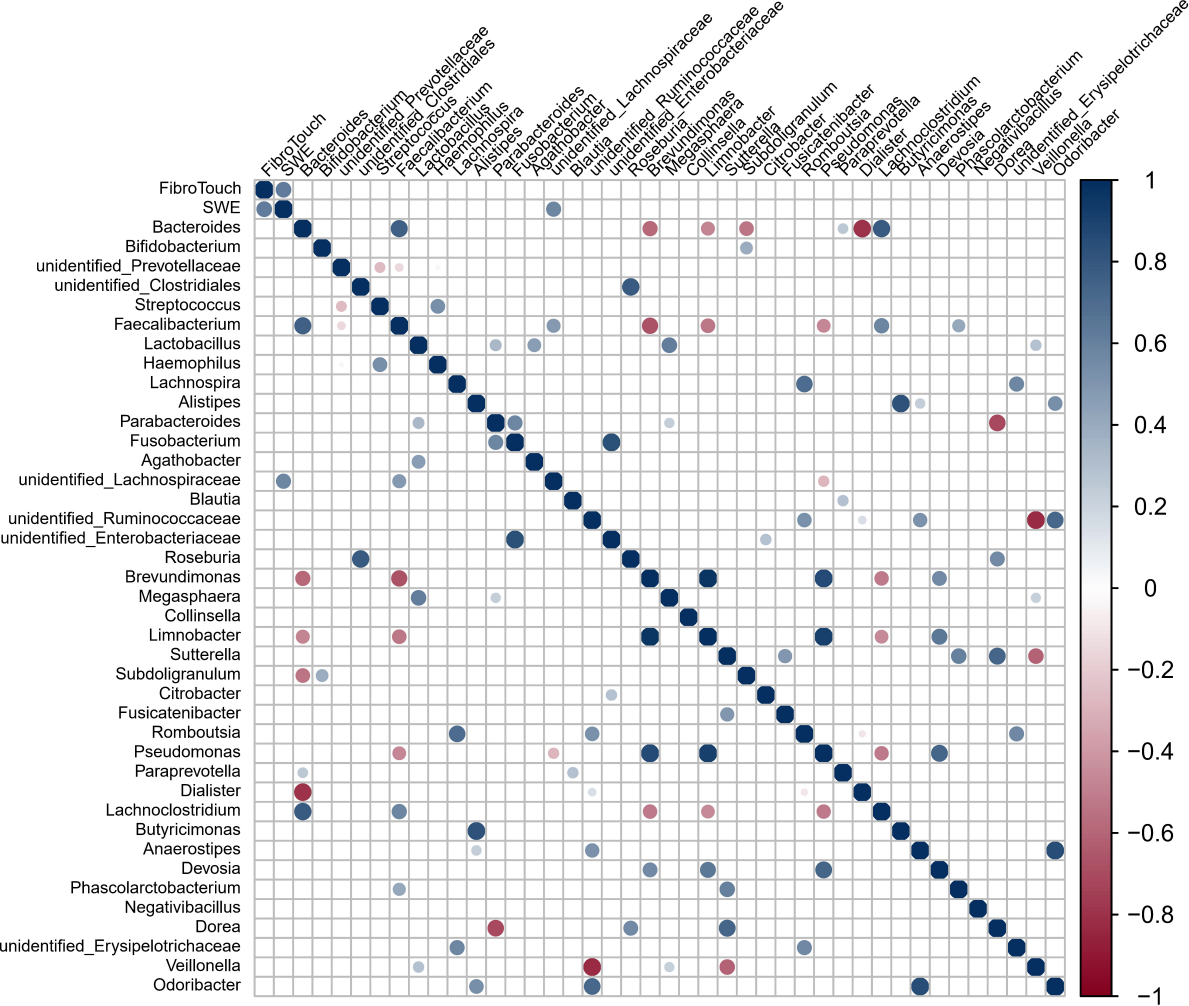
Spearman correlation for variables during fibrosis disease (induced by non-parasitic factors, hepatitis B virus) in humans (n = 12) was shown. Information of participants of study on liver fibrosis induced by non-parasitic factors wes shown in **Table S1**. The methods of FibroTouch and SWE were clinical evaluation strategies in humans. The circle size indicates the magnitude of the correlation. The circle in color indicates *P* < 0.05, *P* < 0.01 or *P* < 0.001.

Based on the results from gut microbiomes in mice and humans, three major fibrosis- or granulomas-related taxa, *Bacteroides*, *Blautia*, and *Enterococcus*, were found to be associated with the level of liver injuries induced by *S. japonicum* and could be used for distinguishing *S. japonicum* infection in humans (**Figures 3**, **4**). To assess the utility of these gut microbes for indicating *S. japonicum* infection, the area under the receiver-operating characteristic curve (AUC) was analyzed (**Figure 5**). The ratio of *Bacteroides* could help discriminate between *S. japonicum-*infected patients and normal with an AUC of 0.8385 and it is 0.9639 for the diagnosis of *S. japonicum-*infected and uninfected mice (**Figure 5A**). The values of AUC generated by predictor *Blautia* of gut microbiota in humans and mice were 0.8182and 0.8478, respectively (**Figure 5B**). *Enterococcus* could be used for distinguishing the granulomatous or fibrosis level in *S. japonicum-*infected patients with a rate of 0.8438, but it could be inefficiently used in mice with a lower AUC value (**Figure 5C**). In addition, The values of AUC generated by the combination of gut microbes could be used for the prediction of schistosomiasis, with a high value of AUC (**Tables 1**, **S2, S3**). However, none of these gut microbiota features was associated with liver injuries induced by the non-parasitic factor (hepatitis B virus, HBV) in humans (**Figure 6**).

**Table 1:** Receiver-operating characteristic (ROC) curves for the diagnosis of liver injuries using the combination of gut microbes.

Combination^a^	Humans		Mice	
	AUC	Significance^b^	AUC	Significance^b^
*Bacteroides*+*Blautia*	0.8308	0.007684	0.965	< 0.0001
*Bacteroides*+*Enterococcus*	0.8385	0.006379	0.9446	< 0.0001
*Blautia*+*Enterococcus*	0.8701	0.001816	0.8598	0.000623
*Bacteroides*+*Blautia*+*Enterococcus*	0.8308	0.007684	0.9538	< 0.0001

a The rate of identified outliers of the combination of bacteria is 10%.

b P ^<^0.05 indicates statistically significant.

## Discussion and Conclusions

Schistosomiasis is still one of the most prevalent neglected tropical diseases worldwide (1). *S. japonicum* infection could induce a variety of fibrotic diseases and lead to gut microbiota dysbiosis (18, 20, 41). Previous studies have demonstrated that the gut microbiota plays an important role in the progress of hepatic diseases *via* the gut-liver axis (25, 42, 43). These studies suggested that gut microbiota are reliably predicted non-invasive biomarkers for the detection of hepatic fibrosis and damage (25–27). However, few studies explored the relationship between the gut microbiota and the level of hepatic injuries induced by *S. japonicum*. Here, we found significant differences in diversities and composition of gut microbiota of mice infected with *S. japonicum* or without. We revealed that gut microbes such as *Bacteroides*, *Blautia*, *Enterococcus*, and *Mucispirillum* displayed a significant correlation with the level of hepatic granuloma, fibrosis, hydroxyproline, ALT or AST in *S. japonicum* infection-induced disease. Furthermore, novel gut microbiota-derived features *Bacteroides*, *Blautia*, and *Enterococcus* and their combinations could be considered as potential biomarkers for detecting liver injuries induced by *S. japonicum* infection in humans and mice. In total, more non-invasive tools for the prediction of *S. japonicum* infection-induced liver injuries may be beneficial to schistosomiasis control.

Liver injuries and alterations of gut microbiota are associated with the infection of schistosomes and other pathogenic or non-pathogenic factors. These factors, such as *S. japonicum* (20, 29, 44), *S. mansoni* (19, 21, 22), *Clonorchis sinensis* (45), HBV (46, 47), hepatitis C virus (HCV) (48), Tetrachloromethane (CCl4) (49) and alcohol (50), induce the dysbiosis of the gut microbiota of animals and humans and acute or chronic liver injuries such as advanced schistosomiasis, liver cirrhosis and liver cancer. Our study found significant differences in the gut microbiota of mice and humans infected with *S. japonicum*, and *S. japonicum* infection showed a decrease in gut microbial diversity of hosts and an increase in the relative abundance of the genera *Bacteroides*, *Blautia* and *Lactobacillus*. A previous study suggested that the alpha-diversity of intestinal flora in liver fibrosis mice is lower than that in normal mice induced by CCl4 and suggested the decline in *Lactobacillus* in the liver fibrosis mice with NOX4 or RhoA intervention (49). In addition, *Blastocystis* infection induced the decrease of *Bacteroides* in children (51). The abundance of genus *Enterococcus* was significantly downregulated in the intestinal tract of mice with inflammatory bowel disease (IBD) after *Schistosoma* soluble egg antigen (SEA) intervention (44) and was associated with the progression of the HBV related acute-on-chronic liver failure (52). Phyla *Firmicutes* and *Bacteroidetes* represent more than 50% of the total bacterial composition in mice infected with *Schistosoma mansoni* (19), which was similar to our findings, However, there is no significant difference in the relative abundance of genera *Bacteroides*, *Blautia* and *Lactobacillus* after *Schistosoma mansoni* infection. In short, pathogenic factors, as well as non-pathogenic factors, could induce gut microbiota dysbiosis, however, it showed differences in the diversity, abundance or species of gut microbiota of hosts.

It is important to reveal the cause-and-effect relationship between gut microbiota and liver diseases. Previous studies have suggested that gut flora affects cirrhosis (53, 54) and fecal microbiota transplantation (FMT) provides new insights into the interactive mechanism between gut microbiota and liver diseases (54–57). However, the causal association between gut flora and schistosomiasis is unclear, and it deserves further studies.

Although our study has added influence factors such as probiotic supplements (29) that may affect the gut microbiota of mice, there were significant differences in diversities and community structures of gut microbiota between *S. japonicum*-infected and control mice. Our study revealed significant changes in gut bacterial diversity and communities of mice infected with different numbers of *S. japonicum* or in different stages. These results were similar to the previous studies (20, 29, 58). In addition, we obtained and analyzed the gut bacterial data from the previous study (30), and we found significant differences in the compositions of gut microbiota of *S. japonicum* infection-induced patients when compared with the control healthy individuals. Overall, our findings revealed that *S. japonicum* infection could significantly shape the compositions of gut microbiota in both mice and humans.

Previous studies have revealed that gut microbiota played an important role in the liver diseases induced by abiotic factors *via* the gut-liver axis and can be considered as biomarkers to distinguish the level of liver injuries (26, 27, 43). However, whether gut microbiota could be defined as a good biomarker of *S. japonicum* infection-induced liver injuries is controversial. Previous studies have indicated that gut bacterial taxa cannot be used as non-invasive biomarkers for *S. japonicum* infection-induced liver cirrhosis (18), and the researchers suggested that the gut microbiota of *S. japonicum* infection-induced liver cirrhosis patients was similar to that of healthy individuals. But our findings and previous studies (20, 30) revealed that *S. japonicum* infection induced significant changes in the compositions of gut microbiota in both mice and humans. In addition, infection of schistosomes, such as *Schistosoma mansoni* and *Schistosoma haematobium*, is associated with the shifts of gut microbiota in rodent models (21, 59) and humans (19, 41, 60–62). These studies implied that there was a strong relationship between gut microbiota features and schistosome infection-induced diseases in mammals.

Fibrosis-related *Ruminococcaceae* and *Veillonellaceae* species could be used to evaluate their effects on liver damage in mammalian NAFLD models (25). A total of 37 bacterial species, such as *Bacteroides caccae*, *Bacteroides dorei* and *Blautia* sp., were used to construct a random forest classifier model to distinguish mild/moderate NAFLD from advanced fibrosis in humans (26). In our study, a range of gut microbes was identified to distinguish the *S. japonicum* infection in mammals using the random forest classifier model, LEfSe and STAMP analysis. Importantly, we further detected the relationship between these gut bacteria and liver injuries using the Spearman correlation. In total, we found that three gut microbes including *Bacteroides*, *Blautia* and *Enterococcus* and their combinations may define as possible predictors for non-invasive detection of liver injuries of schistosomiasis in mammals (humans and mice) but not for hepatic fibrosis diseases induced by non-parasitic factors in humans, which may highlight the specificity of predicted models.

Enterotypes in the landscape of gut microbial community composition (63) show that *Bacteroides* is one of the important gut microbes to distinct community composition types termed enterotypes (42). *Enterococcus*, a gut pathobiont harboring both humans and mice, is associated with liver disease (64). In addition, *Blautia*, a genus of *Firmicutes* with potential probiotic properties that occurs widely in the feces and intestines of mammals (65), is lessened in chronic liver diseases and hepatocellular carcinoma patients (66). These studies have revealed that *Bacteroides*, *Blautia* and *Enterococcus* were significantly associated with biological aspects of humans and animals. Our findings found these gut microbes with potential predictor properties.

Furthermore, our study identified a range of gut bacterial features (*Bacteroides*, *Blautia* and *Enterococcus*) to distinguish schistosomiasis from hepatic injuries and first showed a significant correlation with the level of hepatic granulomas, fibrosis, hydroxyproline, ALT or AST in *S. japonicum* infection-induced disease. Previous studies have revealed the association between gut microbiota and schistosomiasis (20, 29, 30), but did not show more details about the relationship between serologic detection and the level of hepatic injuries in schistosomiasis. Our findings provided more evidence for novel gut microbiota-derived features for non-invasive detection of schistosomiasis.

We acknowledged several limitations of this study. First, to distinguish the fibrosis-specific microbiome, we investigated the relationship between gut microbiota and liver fibrosis using 16S rRNA sequencing but not metagenomics as in the previous study (26). This limited our further investigation to the species level of gut microbiota. Second, although we used gut bacterial data from human individuals in the present study (30), our study lacks cross-sectional studies to allow us to further analyze the relationship between the stool microbiota and disease in schistosomiasis from humans. Nevertheless, our findings were sufficient to confirm the differences in gut microbiota changes according to hepatic injuries and revealed a range of gut microbiota features for non-invasive detection of schistosomiasis. However, it needs further study.

In total, we concluded that the gut microbiome profile changed significantly in *S. japonicum* infection-induced disease and provided preliminary evidence of an association between gut microbiota and hepatic injuries in schistosomiasis. We have described several gut microbiota features and combinations as non-invasive biomarkers for diagnosing liver injuries in mammals with *S. japonicum* infection. Further studies are needed.

## Data Availability Statement

The data presented in the study are deposited in the GenBank repository, accession number (PRJNA836144). The referenced datasets can be found in GenBank repository (accession numbers PRJNA759360 and PRJNA625383) shown in previous studies.

## Ethics Statement

The experiments on participants were reviewed and approved by the Medical Research Ethics Committee of The Third Affiliated Hospital in Sun Yat-sen University ([2019]02-424-01). The patients/participants provided their written informed consent to participate in this study. The animal experiments were reviewed and approved by the Institutional Animal Care and Use Committee of Sun Yat-sen University (Permit No: 2016-104) and the Medical Research Ethics Committee of Sun Yat-sen University (SYSU-IACUC-2019-B517).

## Author Contributions

XYW, XS, ZDW and DL conceived and designed the study. DL and QS carried out the experiments and handled the statistical analysis. DL prepared the interpretation of the data and the original draft. DL, JL, FC and YZ critically revised the draft version of the paper. All authors contributed to the article and approved the submitted version.

## Funding

This work was supported by the National Key R&D Program of China (Nos.2021YFC2300800, 2021YFC2300801, 2020YFC1200100 and 2016YFC1200500), the Natural Science Foundation of Guangdong Province (Nos. 2019A1515012068, 2020A1515010896 and 2021A1515010976), the National Natural Science Foundation of China (Nos. 82161160343 and 82002168), the Fundamental Research Funds for the Central University (No. 22qntd4813), the 111 Project (No. B12003) and the 6th Nuclear Energy R&D Project (No. 20201192). The funders had no role in study design, data collection and analysis, decision to publish, or preparation of the manuscript.

## Conflict of Interest

The authors declare that the research was conducted in the absence of any commercial or financial relationships that could be construed as a potential conflict of interest.

## Publisher’s Note

All claims expressed in this article are solely those of the authors and do not necessarily represent those of their affiliated organizations, or those of the publisher, the editors and the reviewers. Any product that may be evaluated in this article, or claim that may be made by its manufacturer, is not guaranteed or endorsed by the publisher.

## Abbreviations

OTU, operational taxonomic unit; PZQ, Praziquantel; AST, aspartate aminotransferase; ALT, alanine aminotransferase; LSM, liver stiffness measurement; 2D SWE, two-dimensional shear wave elastography; PLS-DA, partial least squares discrimination analysis; PCoA, Principal Co-ordinates Analysis; ANOSIM, analysis of similarities; ROC, receiver-operating characteristic; AUC, area under the ROC curves; STAMP, Statistical analysis of metagenomic profiles; LDA, Linear discriminant analysis; LEfSe, Linear discriminant analysis effect size; PCR, Polymerase chain reaction; HBV, Hepatitis C virus; HCV, Hepatitis C virus; CCl4, Tetrachloromethane; IBD, Inflammatory bowel disease; SEA, *Schistosoma* soluble egg antigen; FMT, fecal microbiota transplantation.
