# Corrigendum: Potential gut microbiota features for non-invasive detection of schistosomiasis

**DOI:** 10.3389/fimmu.2022.998528

**Published:** 2022-08-05

**Authors:** Datao Lin, Qiuyue Song, Jiahua Liu, Fang Chen, Yishu Zhang, Zhongdao Wu, Xi Sun, Xiaoying Wu

**Affiliations:** ^1^ Department of Parasitology, Zhongshan School of Medicine, Sun Yat-sen University, Guangzhou, China; ^2^ Key Laboratory of Tropical Disease Control, Ministry of Education, Guangzhou, China; ^3^ Chinese Atomic Energy Agency Center of Excellence on Nuclear Technology Applications for Insect Control, Provincial Engineering Technology Research Center for Diseases-Vectors Control, Guangzhou, China; ^4^ Department of Clinical Laboratory, Xiangyang No.1 People’s Hospital, Hubei University of Medicine, Xiangyang, China; ^5^ School of Medicine, South China University of Technology, Guangzhou, China; ^6^ The Third Affiliated Hospital, Sun Yat-sen University, Guangzhou, China

**Keywords:** *Schistosoma japonicum*, schistosomiasis, parasites, gut microbiota, non-invasive

In the published article, there was an error in **Figure 1** as published. The description of the Y-axis in **Figure 1A** was incorrectly described. The corrected **Figure 1** appears below.

**Figure 1:**
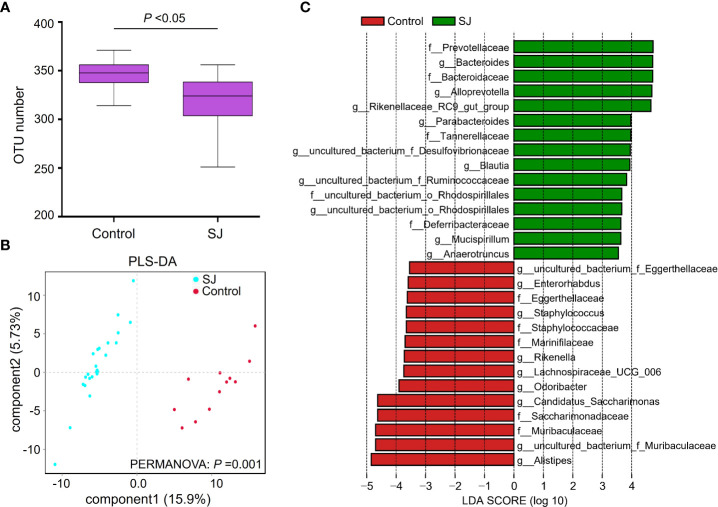
Comparisons of gut microbiota between *S. japonicum*-infected (n = 24) and uninfected (n = 12) mice. **(A)** OTUs analysis. **(B)** PLS-DA analysis. **(C)** Differential gut bacterial taxa were analyzed by LEfSe analysis with LDA score >3.5 between groups. Control: without *S. japonicum* infection mice. SJ; *S. japonicum*-infected mice. *P < 0.05 indicates significant difference.

In the published article, there was an error in the **Funding** statement. The grant number was incorrect. The correct **Funding** statement appears below.

“This work was supported by the National Key R&D Program of China (Nos. 2021YFC2300800, 2021YFC2300801, 2020YFC1200100 and 2016YFC1200500), the Natural Science Foundation of Guangdong Province (Nos. 2019A1515012068, 2020A1515010896 and 2021A1515010976), the National Natural Science Foundation of China (Nos. 82161160343 and 82002168), the Fundamental Research Funds for the Central University (No. 22qntd4813), the 111 Project (No. B12003) and the 6^th^ Nuclear Energy R&D Project (No. 20201192). The funders had no role in study design, data collection and analysis, decision to publish, or preparation of the manuscript”.

The authors apologize for these errors and state that this does not change the scientific conclusions of the article in any way. The original article has been updated.

## Publisher’s note

All claims expressed in this article are solely those of the authors and do not necessarily represent those of their affiliated organizations, or those of the publisher, the editors and the reviewers. Any product that may be evaluated in this article, or claim that may be made by its manufacturer, is not guaranteed or endorsed by the publisher.

