# Supplementary material for: Potential Gut Microbiota Features for Non-Invasive Detection of Schistosomiasis
Source: Front Immunol. 2022 Jul 14;13:941530. doi: 10.3389/fimmu.2022.941530 (PMC9330540; doi:10.3389/fimmu.2022.941530)
Supplement: Supplementary file 1 [file DataSheet_1.pdf]

Supplementary information for

Potential gut microbiota features for non-invasive detection of schistosomiasis

Datao Lin[123#*], Qiuyue Song[124#], Jiahua Liu[12], Fang Chen[5], Yishu Zhang[12], Zhongdao Wu[123], Xi Sun[12*] and Xiaoying Wu[26*]

[1]Department of Parasitology, Zhongshan School of Medicine, Sun Yat-sen University, Guangzhou, China. [2]Key Laboratory of Tropical Disease Control, Ministry of Education, Guangzhou, China. [3]Chinese Atomic Energy Agency Center of Excellence on Nuclear Technology Applications for Insect Control, Provincial Engineering Technology Research Center for Diseases-vectors Control, Guangzhou, China. [4]Department of Clinical Laboratory, Xiangyang No.1 People's Hospital, Hubei University of Medicine, Xiangyang, China. [5]School of Medicine, South China University of Technology, Guangzhou, China. [6]The Third Affiliated Hospital, Sun Yat-sen University, Guangzhou, China.

[#]These authors contributed equally to this work.

[*]Corresponding:

wuxy227@mail.sysu.edu.cn, sunxi2@mail.sysu.edu.cn, lindt5@mail.sysu.edu.cn

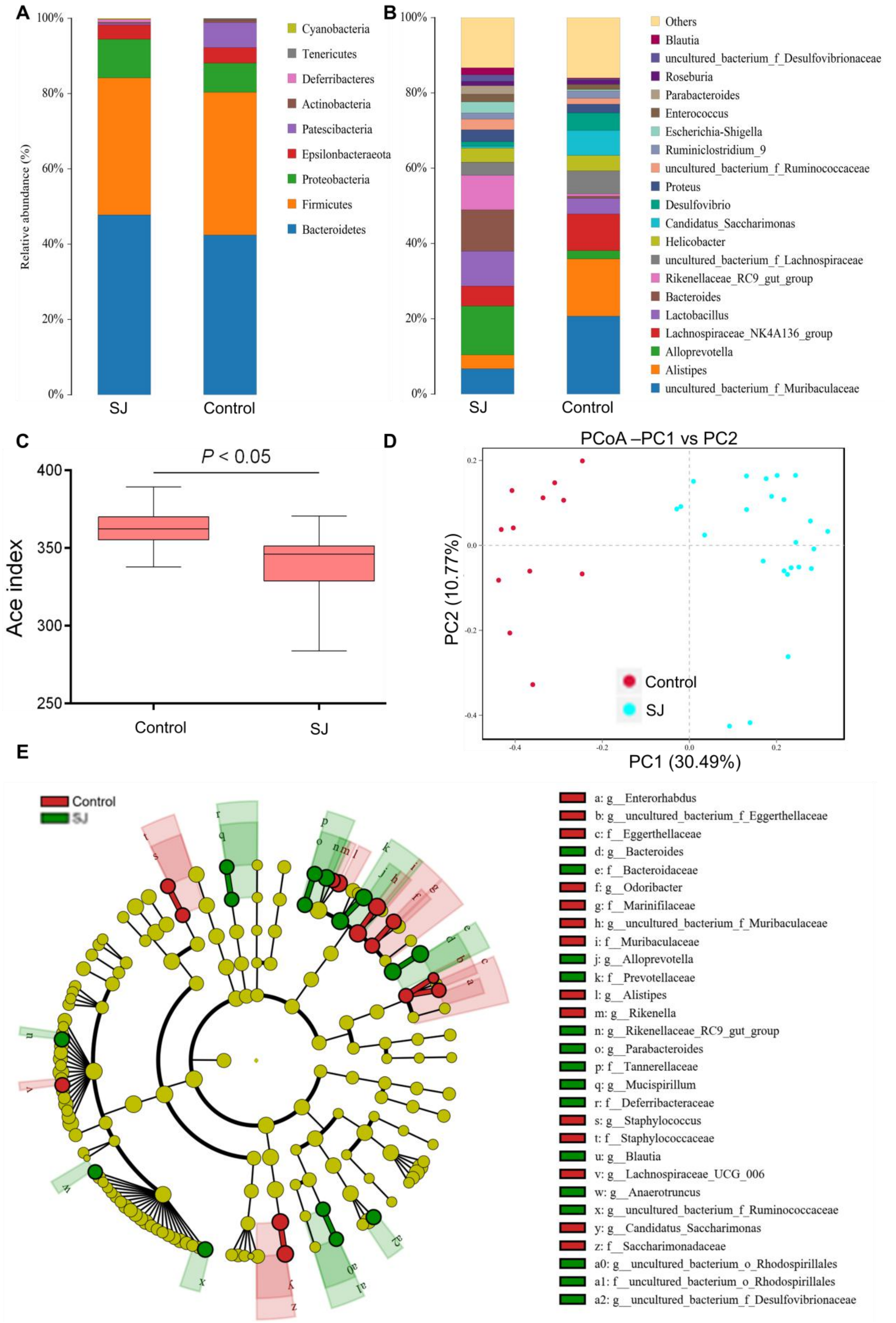

Supplementary Figure S1 Comparisons of gut microbiota between *S. japonicum*-infected and uninfected mice. (A) Phylum. (B) Genus. (C) ACE index. (D) PCoA analysis. (E) Differential gut bacterial taxa were analyzed by LEfSe analysis with LDA score >3.5 between groups. Control: without *S. japonicum* infection mice. SJ: *S. japonicum*-infected mice. *P < 0.05 indicates significant difference.

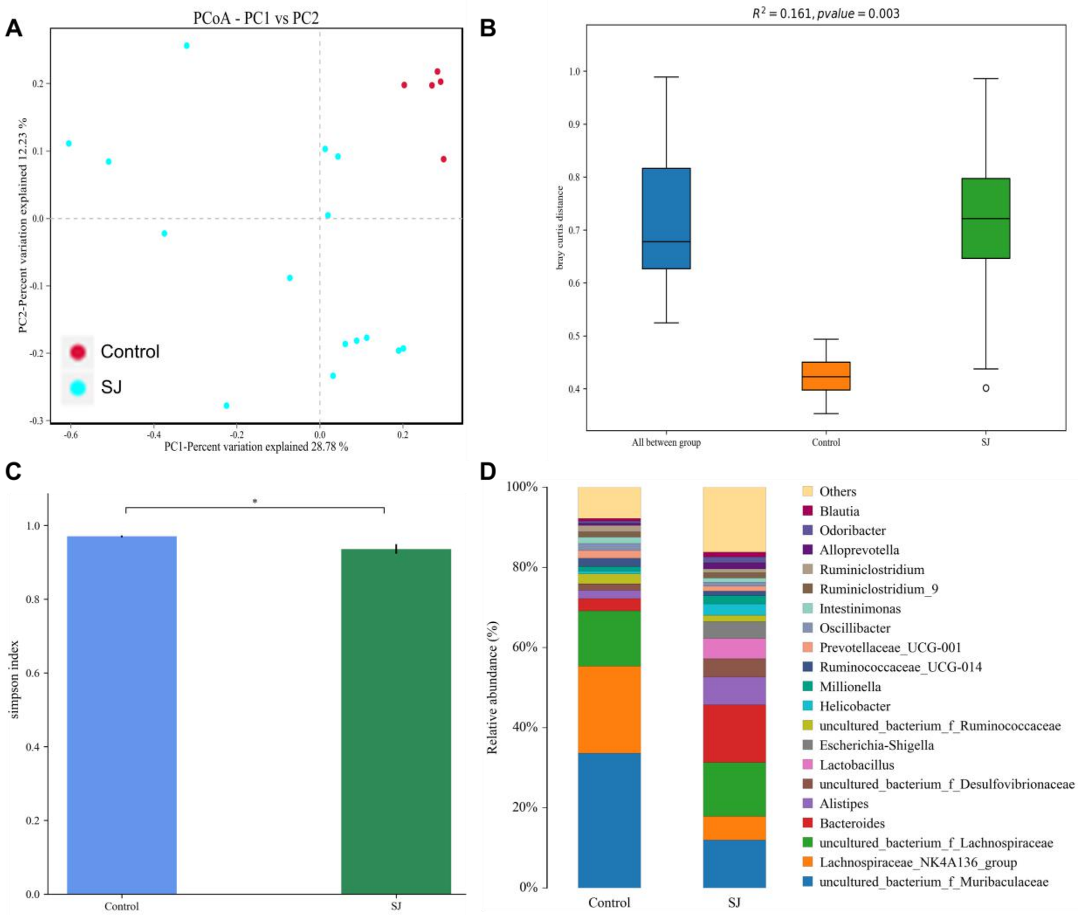

Supplementary Figure S2 Differences in the gut microbiota between *S. japonicum*-infected (n=15) and uninfected (n=5) mice infected with different numbers of *S. japonicum* infection. (A) PCoA analysis. (B) The PERMANOVA analysis. (C) Simpson index. (D) Top 20 genera of gut microbes. Control: without *S. japonicum* infection mice. SJ: *S. japonicum*-infected mice. *$P < 0.05$ indicates significant difference.

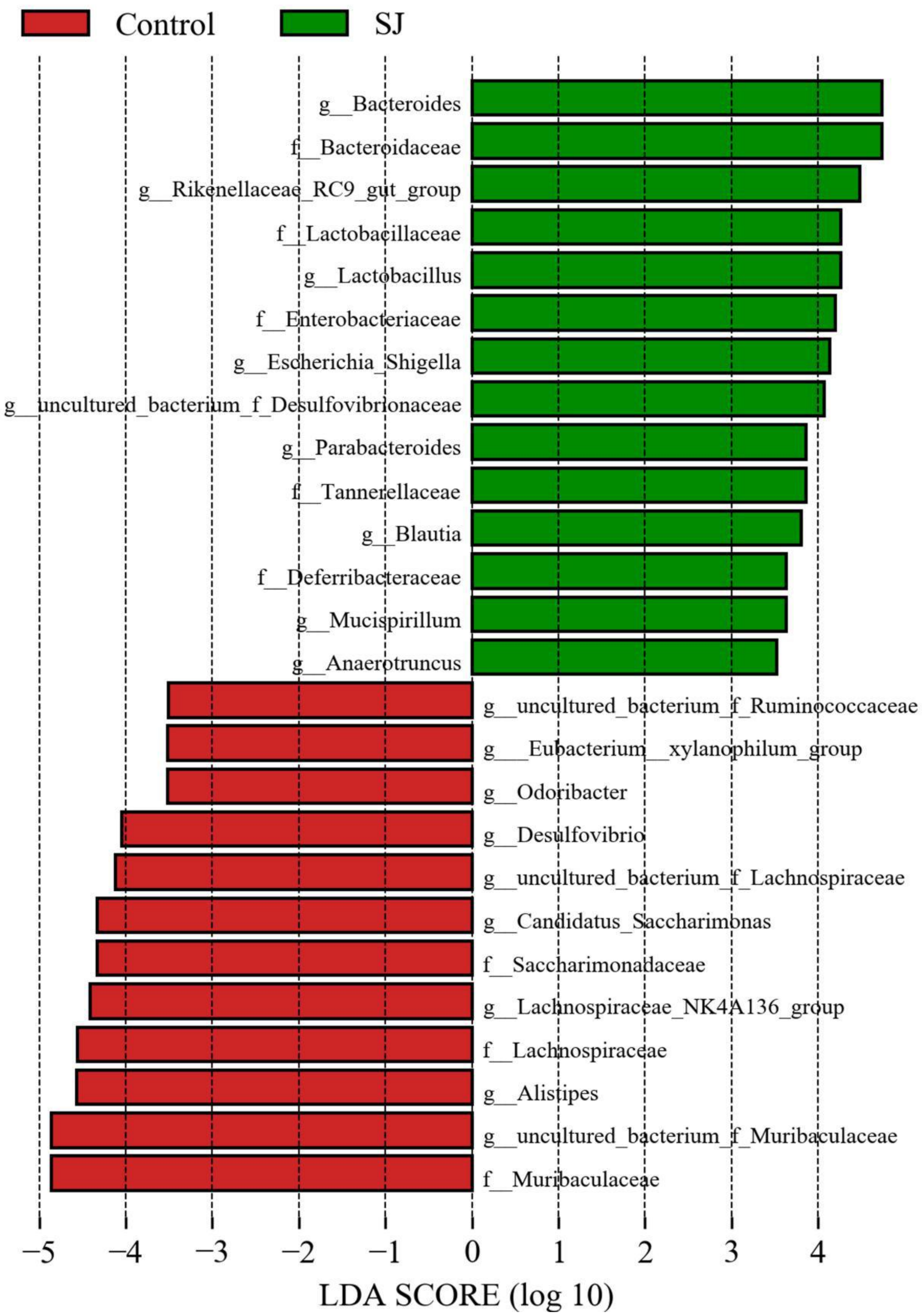

Supplementary Figure S3 Differential gut bacterial taxa in mice were analyzed by LEfSe analysis with LDA score >3.5 between groups. Control: without *S. japonicum* infection mice. SJ: *S. japonicum*-infected mice.

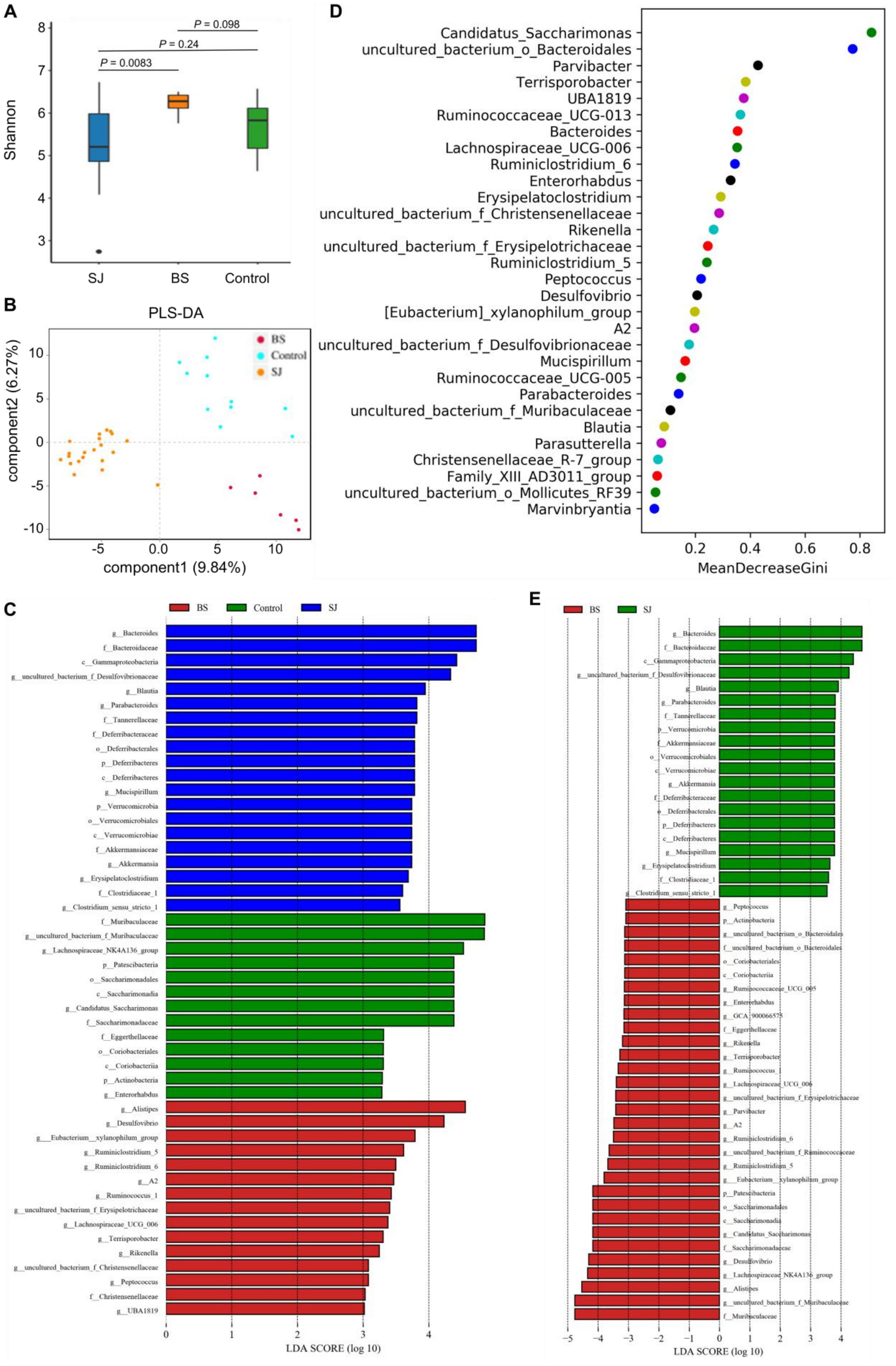

Supplementary Figure S4 Differences in the gut microbiota among groups amd identification of gut microbial biomarkers to distinguish *S. japonicum*-infected _and *Bacillus subtilis*-infected mice. (A) Shannon index. (B) PLS-DA analysis. (C) Differential gut bacterial

taxa were analyzed by LEfSe analysis with LDA score >3 among groups. (D) Top 30 of different gut microbes between SJ and BS groups are shown. (E) Differential gut bacterial taxa were analyzed by LEfSe analysis with LDA score >3 between groups. Control: control mice without *S. japonicum* or *Bacillus subtilis* infection (n=11). SJ: *S. japonicum*-infected mice (n=21) \. BS: *Bacillus subtilis*-infected mice (n=6).

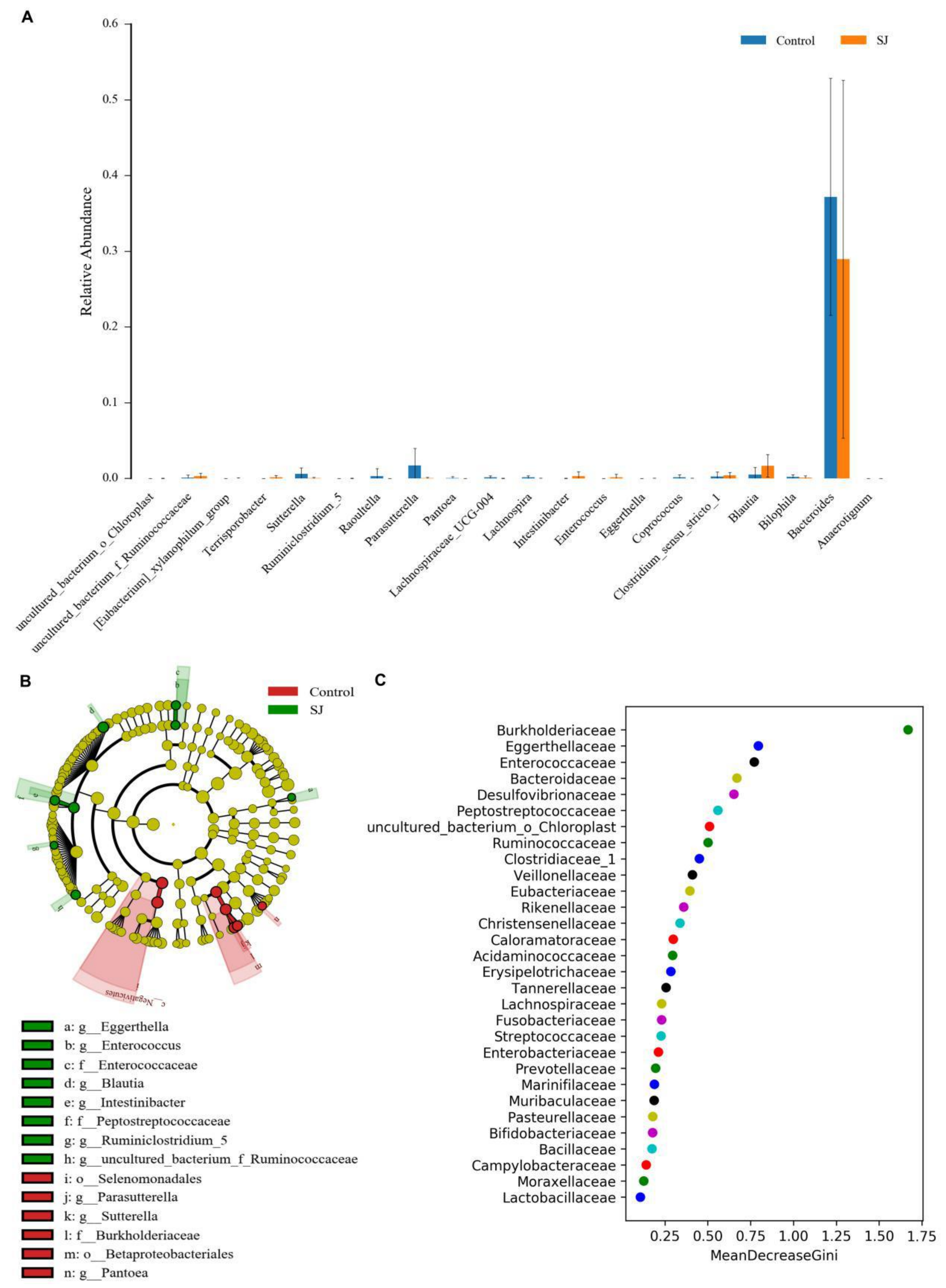

Supplementary Figure S5 Identification of gut microbial biomarkers in *S. japonicum*-infected and uninfected humans. (A) ANOVA analysis. (B) Differential gut bacterial taxa were analyzed by LEfSe analysis with LDA score >3 between groups. (C) Top 30 of different gut microbes between populations are shown. Control: without *S. japonicum* infection humans. SJ: *S. japonicum*-infected humans.

Supplementary Table S1 information of participants of study on liver fibrosis induced by non-parasitic factors in **Fig. 6**. Second liver two half-and-half detect provided reliable clinical test results.

ID	Age	Sex	Diagnosis	Medication	Length of infection	HBsAg	HBsAb	HBeAg	HBeAb	HBcAb
917	68	Male	Hepatitis B virus-induced liver cirrhosis	Entecavir	≧30year	Positive	Negative	Negative	Positive	Positive
935	32	Male	Hepatitis B virus-induced liver cirrhosis	Tenofovir fumarate	≧30year	Positive	Negative	Negative	Positive	Positive
967	35	Male	Hepatitis B virus-induced liver cirrhosis	Tenofovir fumarate	≧30year	Positive	Negative	Negative	Positive	Positive
939	54	Male	Hepatitis B virus-induced liver cirrhosis	Entecavir	≧30year	Positive	Negative	Negative	Positive	Positive
970	60	Male	Hepatitis B virus-induced liver cirrhosis	Entecavir	≧30year	Positive	Negative	Negative	Negative	Positive
915	47	Male	Hepatitis B virus-induced liver cirrhosis	Entecavir	≧30year	Positive	Negative	Negative	Negative	Positive
924	57	Male	Hepatitis B virus-induced liver cirrhosis	Entecavir	≧30year	Positive	Negative	Negative	Positive	Positive
931	57	Male	Hepatitis B virus-induced liver cirrhosis	Entecavir	≧30year	Positive	Negative	Negative	Positive	Positive
933	43	Female	Hepatitis B virus-induced liver cirrhosis	Entecavir	≧30year	Positive	Negative	Negative	Positive	Positive
953	51	Male	Hepatitis B virus-induced liver cirrhosis	Entecavir	≧30year	Positive	Negative	Negative	Negative	Positive
954	51	Male	Hepatitis B virus-induced liver cirrhosis	Entecavir	≧30year	Positive	Negative	Negative	Positive	Positive
957	51	Male	Hepatitis B virus-induced liver cirrhosis	Tenofovir fumarate	≧30year	Positive	Negative	Negative	Positive	Positive

Supplementary Table S2 Receiver-operating characteristic (ROC) curves for the diagnosis of liver injuries using the combination of gut microbes.

Combination[c]	Humans		Mice	
	AUC	Significance[d]	AUC	Significance[d]
Bacteroides+Blautia	0.6727	0.1392	0.939	< 0.0001
Bacteroides+Enterococcus	0.6788	0.1258	0.902	< 0.0001
Blautia+Enterococcus	0.8182	0.006457	0.719	0.01545
Bacteroides+Blautia+Enterococcus	0.6667	0.1536	0.9107	< 0.0001

[c]The rate of identified outliers of the combination of bacteria is 0%

[d]P <0.05 indicates significant.

Supplementary Table S3 Receiver-operating characteristic (ROC) curves for the diagnosis of liver injuries using the combination of gut microbes.

Combination[e]	Humans		Mice	
	AUC	Significance[f]	AUC	Significance[f]
Bacteroides+Blautia	0.7552	0.03451	0.965	< 0.0001
Bacteroides+Enterococcus	0.8385	0.006379	0.9371	< 0.0001
Blautia+Enterococcus	0.8701	0.001816	0.8287	0.00134
Bacteroides+Blautia+Enterococcus	0.7552	0.03451	0.9457	< 0.0001

[e]The rate of identified outliers of the combination of bacteria is 5%

[f]P <0.05 indicates significant.
